# Implementation of Nurse Prescriptions throughout the Public Health System in Catalonia (2021–2022)

**DOI:** 10.3390/healthcare12121232

**Published:** 2024-06-20

**Authors:** Rosa Bayot i Escardívol, Enric Mateo-Viladomat, Paola Galbany-Estragués, Mariona Vilar-Pont, Miquel Angel Calderó i Solé, Gerard Mora-López, Raquel Flores-Montoya, Montse Vicente-Belis, Núria Escoda-Geli, Andrea Molina-Nadal, Olga Canet-Vélez, Glòria Jodar-Solà

**Affiliations:** 1 Official College of Nurses of Barcelona, 08019 Barcelona, Spain; rbayot@coib.cat (R.B.i.E.); emateo@coib.cat (E.M.-V.); pgalbany@coib.cat (P.G.-E.); gjodar@coib.cat (G.J.-S.); 2 Department of Fundamental and Clinical Nursing, Faculty of Nursing, University of Barcelona, 08907 L’Hospitalet de Llobregat, Spain; 3 Official College of Nurses of Girona, 17004 Girona, Spain; vocal3@coigi.cat; 4 Official College of Nurses of Lleida, 25008 Lleida, Spain; macaldero@coill.org; 5 Official College of Nurses of Tarragona, 43003 Tarragona, Spain; gmora@codita.org; 6 Catalan Health Service (CatSalut), Catalan Department of Health, 08028 Barcelona, Spain; raquelflores@catsalut.cat (R.F.-M.); mvicente@catsalut.cat (M.V.-B.); nescoda@catsalut.cat (N.E.-G.); amolinan@catsalut.cat (A.M.-N.); 7 Global Health, Gender and Society (GHenderS), Blanquerna School of Health Sciences, Ramon Llull University, 08025 Barcelona, Spain

**Keywords:** nurse prescribing, medication, health products, implementation, electronic prescription

## Abstract

Background: Nurses in Catalonia have always prescribed health advice, health products, and medication in line with the professional competency of the discipline. Legislation about nurse prescriptions and the implementation of nurse prescribing varies widely among different countries. This article reports data regarding nurse prescribing in Catalonia in 2021 and 2022. Methods: This retrospective longitudinal study analyzed data from all care-providing units in Catalonia’s integrated public health system. Results: The number of nurse prescriptions increased from 139,435 in 2021 to 573,822 in 2022, and the number of nurses issuing prescriptions increased from 3604 in 2021 to 5563 in 2022. The proportion of prescriptions for different products was similar in the two years analyzed. Prescriptions for medication increased by 7.5% in 2022. Conclusions: Nurse prescribing is a recent advance in Catalonia. Despite some difficulties in rollout, the data indicate that this practice is becoming consolidated, as in other European countries.

## 1. Introduction

Nurse prescribing enables nurses to use clinical criteria and good judgment to select and indicate techniques, health products, and drugs to meet the needs of the patients they treat [1]. Nurse prescribing has been implemented in increasingly more countries. Current legislation in the United Kingdom allows duly trained and accredited nurses and pharmacists to prescribe treatments according to two models: independent prescription and complementary prescription. In the independent prescription model, nurses have access to the entire British National Formulary [2]; in other words, using specific criteria and protocols, they can prescribe any of the drugs contained therein. Nurses in the United Kingdom were the pioneers in nurse prescribing (NP). They also started with a restricted list of products, similar to NP in Catalonia (Spain) in community care. A study by Ruiz et al. has shown that NP practice is associated with higher quality care for people requiring medical services, more efficient use of time and resources, and better collaboration among healthcare professionals [3]. NP is described as a process that increases nurses’ decision-making and leadership capabilities, promoting greater empowerment in the workplace, with expanded recognition of their roles and competencies, becoming references for patients and colleagues [4].

In the last 20 years, many countries have given nurses the legal authority to prescribe, although the legal, practical, organizational, and educational requirements for nurse prescribing vary considerably among countries [5]. Due to the increasing demand for healthcare, the shortage of doctors, the need to improve care and treatment, and to reduce the rising healthcare costs, nurse prescribing has been gaining ground in various countries [6,7]. With different legal restrictions, nurses have the authority to prescribe in many countries, including the United States, Australia, Canada, Finland, New Zealand, Norway, the Netherlands, Sweden, and Spain [8].

In Spain, there are two main models of nurse prescription: autonomous/independent prescribing and collaborative/complementary prescribing [9]. Independent or autonomous prescribing refers to the right of nurses to prescribe medications based on their own decisions for certain predefined medications or diseases. On the other hand, complementary, semi-autonomous prescribing grants nurses the right to prescribe medications according to the patient’s treatment plan or the diagnosis carried out by a physician or another independent prescriber, following protocols and clinical practice guidelines [6]. The two models are not mutually exclusive; both are employed in routine care [10]. The Health Quality and Assessment Agency of Catalonia points to a strong consensus regarding the benefits of nurse prescriptions, including empowering nurses, more patient-centered care, and greater continuity of care [11].

**Figure 1:**
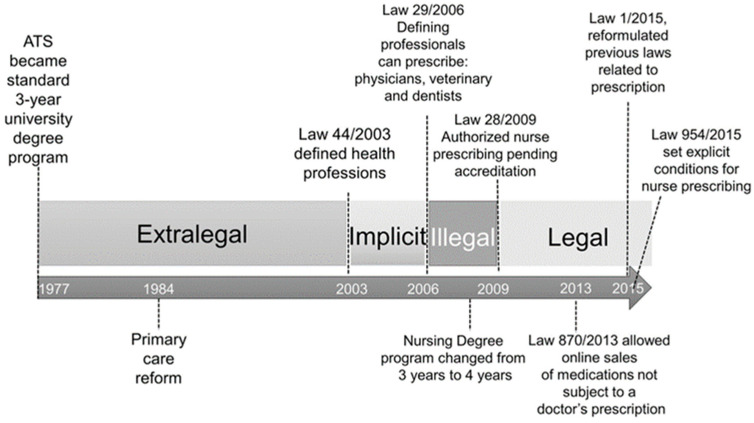
Timeline showing the legal status of nurse prescription in Spain since the democratic transition [15].

The legislation about nurse prescriptions in Spain has been confusing, changing often in the last 40 years. Nurse prescribing existed in a legal void from the beginning of the democratic regime in 1977 until 2003, when the passage of Law 44/2003 [12] regulating the health professions in Spain seemed to imply that nurses could prescribe. However, three years later, Law 29/2006 [13] on the rational use of medication and health products explicitly stated that only physicians and dentists could prescribe drugs. This law was amended three years later (Law 28/2009) [14] to make it clear that nurses could independently prescribe health products and over-the-counter medications. Figure 1 is a timeline showing the legal status of nurse prescription in Spain over time.

Legislation about nurse prescription has also varied widely in other countries. In 2019, some type of nurse prescription was permitted by law in 13 countries: Cyprus, Denmark, Estonia, Finland, France, Ireland, the Netherlands, Norway, Poland, Spain, Sweden, the United Kingdom, and Switzerland [16]. In other countries (e.g., Iran), nurse prescriptions have not been implemented [17].

Nurses in Ireland have played a pioneering role in nurse prescribing, becoming an international reference for the practice. Their experience has been validated by patients’ high opinions of the medications prescribed and of the quality of care [18]. The implementation of nurse prescribing has not always been smooth. It has received support [19], but it has also had to overcome hurdles [20]; moreover, registering prescriptions also increases nurses’ workload [21,22]. In their 2022 review, Edwards et al. [23] list factors that facilitate nurse prescription (e.g., clear roles, support, managerial leadership, clinical protocols and directives, and patient approval [24,25,26,27]), as well as factors that hinder its implementation (e.g., generating prescriptions and electronic registers [28]). On balance, the implementation of nursing prescription has been positive [29], contributing to the development of nurses’ clinical skills [8].

Within Europe, each country decides which drugs are indicated for different processes and how those drugs can be prescribed [16]. In 2018, only 2% of nurses in Ireland were able to prescribe medications [18].

In Catalonia, nurses routinely prescribe health advice, health products (e.g., blood glucose monitors and test strips or products for wound care), and certain medicines (e.g., vaccines, antiseptics, or nonsteroidal anti-inflammatory drugs) based on the professional competencies of the profession; however, the legal framework for these prescriptions remains poorly defined.

In primary care, 20% of patients attending nursing appointments required at least one prescription; 72% of these prescriptions were for medication [30]. At the beginning of 2021, with the deployment of the project integrating nurse prescriptions into the electronic prescription system, around 6.000 nurses in the public health system of Catalonia (SISCAT) applied for digital cards to include their medication and medical product indications in the integrated electronic prescription system (SIRE). In this new context, this study aims to understand how nurses in the SISCAT prescribe medications and medical products after the integration of their therapeutic plans into the integrated electronic prescription system (SIRE). The current study aimed to analyze the implementation of nurse prescription in Catalonia through an integrated electronic prescription system from 2021 through December 2022.

## 2. Materials and Methods

### 2.1. Design

All residents of Catalonia have the right to public healthcare services. This retrospective longitudinal study analyzed the activity in care-providing units of Catalonia’s integrated public health system (SISCAT) registered from May 2021 through December 2022. Our institution’s research ethics committee approved the study (CER-*FCSB #2022-11-02), waiving the need for informed consent because all data were extracted directly from the system’s anonymized pharmaceutical register, making it impossible to identify participants. The study followed the principals laid out in the Helsinki Declaration and complied with Spanish law (LOPD 3/2018) and European directives on data protection (EU 2016/679).

### 2.2. Population

The study population comprised nurse prescribers working in the care-providing units of Catalonia’s public health system, except those who issued no prescriptions and those working at units that issued <20 electronic nurse prescriptions during the study period.

### 2.3. Source of Data

All data were extracted directly from the public-health-system-integrated electronic prescription system, which is designed to integrate the processes of prescribing and dispensing drugs as well as to facilitate coordination among health professionals by making information about treatment plans available in real time. Prescribers use a digital certificate to access the system and to electronically sign the indications for drugs and health products. Patients also have access to their treatment plans, which include the name of the product or drug, dosage, and the foreseen duration of treatment. To carry out prescriptions, accredited nurses require a digital certificate to access and electronically sign the prescription of medications and healthcare products.

### 2.4. Variables and Measuring Instruments

We analyzed the number of electronic nurse prescriptions and the number of nurses that issued these prescriptions in each primary care center of Catalonia’s 10 healthcare regions (territories comprising a neighborhood or district within an urban area or one or more towns in rural areas) and in each administrative area (territorial divisions in which primary care centers and hospitals are grouped for the purpose of operational planning and analyses of flow).

We also analyzed these variables according to the patient’s diagnosis, the health product prescribed, and the Anatomical, Therapeutic, Chemical (ATC) classification for each month and year of the study.

### 2.5. Statistical Analysis

The statistical analysis was conducted as a classic descriptive analysis. Some mean values were calculated, but the focus was primarily on describing frequencies and percentages. The aim was to detail nurse prescriptions across different care provider units. The following descriptive statistics were calculated: the number of nurse prescriptions over different time periods, the average number of nurse prescriptions per patient, the rate of prescriptions for medications or medical products per patient, the percentage of care units where nurse prescriptions were issued, the prescription rate for the entire assigned population, the rate of nurses issuing ≥1 prescription relative to the total number of nurses with digital certification, the percentage of nurse prescriptions out of the total number of prescriptions, and the month-over-month growth in the number of nurse prescriptions in each care unit and for the products that nurses are authorized to prescribe. Therefore, the analysis was purely descriptive, and the statistical software used to analyze the data was Stata.

## 3. Results

A total of 3604 nurses in 2021 and 5563 in 2022 issued electronic prescriptions from the care-providing units that met the inclusion criteria. The data were anonymized, making it impossible to identify the nurses.

### 3.1. Number of Nurse Prescriptions and Number of Nurses Who Issued Prescriptions

The number of nurse prescriptions and the number of nurses who issued prescriptions increased between 2021 and 2022 (Table 1).

In 2021, the rate of nurse prescriptions per healthcare region ranged from 4 to 38 per 100,000 insured persons. In 2022, the number of nurse prescriptions increased in all healthcare regions. In 2021, the healthcare region with the highest percentage of nurses issuing prescriptions (84 prescribing nurses per 100,000 insured persons) (Table 2). In 2022, the number of nurses who issued prescriptions increased in all healthcare regions, and Barcelona city remained the region with the lowest rate of nurses who issued prescriptions (61 prescribing nurses per 100,000 insured persons). In 2022, Terres de l’Ebre was the region with the highest rate of nurses who issued prescriptions (96 prescribing nurses per 100,000 insured persons). In 2021, Camp de Tarragona was the region with the highest mean number of nurse prescriptions per nurse (48), whereas the Alt Pirineu and Aran region was the region with the lowest value for this variable (9). In 2022, the mean number of prescriptions per nurse increased in all healthcare regions. Barcelona city was the region with the highest mean number of nurse prescriptions per nurse (117) (Table 2 and Table 3).

### 3.2. Nurse Prescriptions by Year and Month of Nurse Prescribing

The number of nurse prescriptions increased every month between January 2021 and December 2022, starting out with 176 nurse prescriptions issued in all Catalonia in January 2021 and finishing with 64,758 in December 2022. Likewise, the number of nurses who issued prescriptions increased every month in the study period, starting out with 43 prescribing nurses in all Catalonia in January 2021 and finishing with 4252 in December of 2022. The mean number of prescriptions issued by each nurse increased over time, albeit more modestly and less steadily (Table 4).

### 3.3. Nurse Prescriptions Broken down by Product Prescribed

Products were classified in two categories, namely material (e.g., cotton and sterile dressings, urine collection kits, bandages, ostomy bags, etc.) and medication, which was further classified according to the main organ/system for which they were prescribed (nervous system, digestive system, respiratory system, etc.). The proportion of prescriptions for material was greater in 2021 (73%) than in 2022 (65.5%), and the proportion of prescriptions for medication was lower in 2021 (27%) than in 2022 (34.5%). The proportion of prescribing nurses that issued prescriptions for material was 83.2% in 2021 and 89.9% in 2022, and the proportion of prescribing nurses that issued prescriptions for medication was 76.1% in 2021 and 85.6% in 2022. In both 2021 and 2022, the mean number of prescriptions per nurse was greater for material than for medication (Table 5).

The number of prescriptions was higher in 2022 than in 2021 for all ATCs except the nervous system, which decreased from 66.7% in 2021 to 56.4% in 2022 (Table 6). The nervous system was the ATC that accounted for the greatest proportion of prescriptions in both years (66.7% in 2021 and 56.4% in 2022), followed by the musculoskeletal system (13.7% in 2021 and 16.7% in 2022), the digestive system and metabolism (8.5% in 2021 and 10.4% in 2022), and the respiratory system (7.0% in 2021 and 11.9% in 2022). The remaining ATCs accounted for ≤2% in both years of the study. The number of nurses issuing prescriptions was higher in 2022 than in 2021 for all ATC categories (Table 6).

Prescriptions for material were similar in 2021 and 2022. In both years, the most prescribed materials were cotton and sterile dressings (51.4% in 2021 and 47.6% in 2022), followed by products related to body waste (urine collection kits, external urinary and fecal incontinence devices) (25.4% in 2021 and 28.5% in 2022) and gauze (10.3% in 2021 and 10.0% in 2022). The remaining products accounted for <5% of nurse prescriptions in both years. The number of nurses who issued prescriptions in each category was greater in 2022 than in 2021 (Table 7).

### 3.4. Diagnoses Associated with Nurse Prescriptions

The number of nurse prescriptions for the following diagnoses was higher in 2022 than in 2021:

- Symptoms, signs, and abnormal findings on complementary tests not classified under other categories;
- Genitourinary disease;
- Respiratory disease;
- Certain infectious diseases and parasitic diseases;
- Diseases of the ear and mastoid process.

The diagnosis that resulted in the greatest percentage of nurse prescriptions in both years was “symptoms, signs, and abnormal findings on complementary tests not classified under other categories” (14.6% in 2021 and 15.3% in 2022), followed by “diseases of the skin and subcutaneous tissues” (9.6% in 2021 and 8.4% in 2022) and “traumatic lesions, poisoning, and other external causes” (8% in 2021 and 5.9% in 2022).

## 4. Discussion

Nurse prescriptions for medication and health products have increased around the world, but the heterogeneity of legal and organizational frameworks in different countries makes the analysis of this practice complex [4].

In February 2019, the government of the region of Catalonia set up a system based on digital certificates to enable nurse prescribing [31]. In January and the first two weeks of February 2021, a pilot program for nurse prescriptions was implemented, with the participation of 115 nurses (105 working in primary care and 10 working in specialized care) and of 34 healthcare teams. The program has been progressively expanded to cover the entire region. As in other experiences, we identified factors that facilitated or hindered the implementation process in the preparation, training, transition, and consolidation phases [23].

In our experience, the first two phases were more structured; despite the positive results achieved so far, it is important to ensure that these results have been maintained in the transition phase and will be maintained in the consolidation phase, which will follow the analysis carried out in the present study. In the consolidation phase, the time since the initial deployment and the accumulated experience can facilitate the practice of nurse prescribing [28]. In the current study, the health region with the largest insured population and the first region in which nurse prescriptions were implemented are the regions where the rates of nurse prescriptions and nurse prescribers (per 1000 insured persons) are the highest. As in the United Kingdom, a pioneer in nurse prescriptions, in Spain, one of the priorities is to consolidate nurse prescribing in primary care to optimize the use of medications [32]. In Catalonia, regulating nurse prescriptions provided a legal framework for this practice, which recognizes nurses’ authority and autonomy [4]. In the period analyzed here, electronic prescriptions were a transversal element integrated into the health system including all public healthcare providers.

Our data show that the number of nurse prescriptions quadrupled in one year and the number of nurse prescribers increased by more than 50% in the same period. This increase could be attributed to increased confidence in prescribing and increased opportunities for prescribing [8,32,33]. The total number of nurse prescriptions for medication increased from 37,613 in 2021 to 197,710 in 2022; this huge increase is undoubtedly related to the increase in the number of nurse prescribers during this period. The number of nurses who issued prescriptions in Catalonia increased from 3604 in 2021 to 5563 in 2022. Similar increases were reported in Poland, where the number of nurse prescriptions increased nearly sixfold, and the number of nurse prescribers doubled [34]. A retrospective study in Wales also found that the number of prescribing nurses doubled, and the prescriptions increased greatly after implementation in primary care [35].

Rates of prescription can vary between provinces due to the differences in their populations and in the number of nurse prescribers in the territories [36]. The health regions in the province of Barcelona have the largest insured populations; these regions account for 65% of all nurse prescriptions in both years analyzed. On the other hand, the Terres de l’Ebre region, whose population accounts for only 2.3% of Catalonia, was at the forefront of the rollout and had a high rate of nurse prescriptions in both years of the study. One of the reasons for uneven nurse prescribing in Catalonia could be related to organizational and institutional contexts that can promote or impede the implementation of this practice [37].

Interestingly, in 2021, 50% of all nurse prescriptions were issued in the last quarter of the year; in 2022, although the increase in the number of prescriptions was more regular, the greatest increase was also observed in the last three months. It is noteworthy that the number of nurse prescriptions doubled between January and December of 2022. Although we have identified the factors that could have influenced these trends, it seems that nurse prescribing is becoming consolidated. Healthcare administrators should facilitate nurse prescriptions in daily clinical practice [38]. As nurses gain confidence in prescribing, they are likely to issue more prescriptions [39].

During the first year, 73% of nurse prescriptions corresponded to health products such as bandages, gauze, and urine collection kits; nurses have always guided patients in this area. During the second year, although 85% of the nurse prescriptions issued corresponded to these products, more nurse prescriptions for medication were issued than in the previous year, possibly owing to nurses’ expertise and their demand to expand the catalogue of medications that nurses can prescribe. Other authors have reported a significant increase in nurse prescriptions for medications in patients with chronic disease [34]. Other studies have also found a preponderance of prescriptions for health products [36].

Among nurses working in medical specialties, in both years of the study, nurse prescriptions for medications targeted the nervous/musculoskeletal system first, followed by the digestive system and respiratory system. In Finland and Spain, medications for chronic and acute medication are highly prevalent [16]. In Ireland, the most frequently prescribed medications are analgesics, anti-inflammatory drugs, and vaccines and antibiotics for adults [18]. In the United Kingdom, New Zealand, and the Netherlands, nurse prescribers play an important role in the treatment of older patients with chronic conditions [40].

Nurses in Catalonia consider that the catalogue that they can prescribe is insufficient to enable independent prescription and are asking the authorities to expand it to improve care [4]. Nurse prescribing facilitates professional development and can help redesign primary care to make it more sustainable [22,41].

In 2023, nurses also indicated actions within patients’ treatment programs (e.g., rest requiring time off work); nurses provided indications of this type to 381,496 patients in addition to the 376,112 nurse prescriptions issued that year.

The existence of nurse prescribing has highlighted facilitators in prescribing systems. Some of these include, in New Zealand and the United Kingdom, proper supervision, support from colleagues in the multidisciplinary team, and prescribing education and guidelines [42]. In a study conducted in Spain, similar factors were noted, such as the need for communication and interprofessional and collaborative work, a solid educational foundation in nurse prescribing at the university level, ongoing training, and institutional support [43].

## 5. Conclusions

Nurse prescribing in Catalonia is a relatively recent practice that has presented some challenges in its implementation. Its development has been significant not only in urban areas but also in the rest of the province, particularly in rural areas. Despite these difficulties, nurse prescribing is consolidating, similar to what is observed in other European countries. Regarding patient safety and satisfaction, specific evidence on these aspects in Catalonia is very limited. Although progress in professional accreditation and regulatory adaptation is recognized, no conclusive studies have been found that thoroughly evaluate the safety of nurse prescribing and patient satisfaction in this context. International studies have shown that nurse prescribing improves treatment adherence [44]. As in other countries, information on the safety and benefits of nurse prescribing can help guide large-scale implementation and continuity of this practice in Catalonia [45]. Evidence of safe prescribing with patient satisfaction needs to be included in the evaluation of nurse prescribing in Catalonia.

## 6. Limitations

The present study has certain limitations. Its findings are primarily descriptive and do not allow for strong inferential conclusions. However, they provide essential preliminary insights into the rollout of nurse prescribing in Catalonia. Future research should aim to address these limitations by incorporating inferential statistical methods and longitudinal data.

## Figures and Tables

**Table 1:** Number of nurse prescriptions and number of nurses that issued prescriptions (2021–2022).

Total Catalonia	Nurse Prescriptions		Insured Population		Nurse Prescriptions (per 1000 Insured) **	Prescribing Nurses ***		Prescribing Nurses (per 100,000 Insured) ****	Mean Prescriptions/Nurse *****
	N	%	N	%		N	%		
2021	139,435	100	7,697,069	100	18	3604	-	47	39
2022	573,822	100	7,794,749	100	73,6	5563	-	71	103

** Nurse prescriptions/Insured population × 1000. *** A single nurse could issue prescriptions in different administrative areas. **** Prescribing nurses/Insured population × 100,000. ***** Nurse prescriptions/prescribing nurses.

**Table 2:** Number of nurse prescriptions and number of nurses who issued prescriptions by health region (2021).

2021	Nurse Prescriptions		Insured Population		Nurse Prescriptions (per 1000 Insured) *	Prescribing Nurses **		Prescribing Nurses (per 100,000 Insured) ***	Mean Prescriptions/Nurse ****
Health Region	N	%	N	%		N	%		
Alt Pirineu i Aran	257	0.2	68,518	0.9	4	30	0.8	44	9
Barcelona	84,593	60.7	5,068,716	65.9	17	2208	61.3	44	38
South Metropolitan	23,110	16.6	1,376,702	17.9	17	706	19.6	51	33
North Metropolitan	33,926	24.3	1,996,249	25.9	17	850	23.6	43	40
Barcelona City	27,557	19.8	1,695,765	22.0	16	652	18.1	38	42
Camp de Tarragona	16,590	11.9	614,829	8.0	27	344	9.5	56	48
Central Catalonia	6358	4.6	531,073	6.9	12	295	8.2	56	22
Girona	17,258	12.4	870,481	11.3	20	398	11.0	46	43
Lleida	7516	5.4	365,321	4.7	21	188	5.2	51	40
Terres de l’Ebre	6863	4.9	178,112	2.38	38	149	4.1	84	46
Total Catalonia	139,435	100	7,697,069	100.0	18	3604	-	47	39

* Nurse prescriptions/Insured population × 1000. ** A single nurse could issue prescriptions in different administrative areas. *** Prescribing nurses/Insured population × 100,000. **** Prescribing nurses/Insured population × 100,000.

**Table 3:** Number of nurse prescriptions and number of nurses who issued prescriptions by health region (2022).

2022	Nurse Prescriptions		Insured Population		Nurse Prescriptions (per 1000 Insured) *	Prescribing Nurses **		Prescribing Nurses (per 100,000 Insured) ***	Mean Prescriptions/Nurse ****
Health Region	N	%	N	%		N	%		
Alt Pirineu i Aran	1240	0.2	70,018	0.9	18	45	0.8	64	28
Barcelona	391,260	68.2	5,130,528	65.8	76	3558	64.0	69	110
South Metropolitan	97,358	17.0	1,390,255	17.8	70	1026	18.4	74	95
North Metropolitan	170,458	29.7	2,011,091	25.8	85	1481	26.6	74	115
Barcelona City	123,444	21.5	1,729,182	22.2	71	1051	18.9	61	117
Camp de Tarragona	47,478	8.3	624,163	8.0	76	441	7.9	71	108
Central Catalonia	27,206	4.7	535,969	6.9	51	426	7.7	79	64
Girona	66,018	11.5	883,512	11.3	75	656	11.8	74	101
Lleida	24,745	4.3	369,923	4.7	67	290	5.2	78	85
Terres de l’Ebre	15,875	2.8	180,636	2.3	88	173	3.1	96	92
Total Catalunya	573,822	100	7,794,749	100	74	5563	-	71	103

* Nurse prescriptions/Insured population × 1000. ** A single nurse could issue prescriptions in different administrative areas. *** Prescribing nurses/Insured population × 100,000. **** Prescribing nurses/Insured population × 100,000.

**Table 4:** Number of nurse prescriptions and number of nurses who issued prescriptions in each month of the study period (2021–2022).

	2021					2022				
	Nurse Prescriptions		Prescribing Nurses *		Mean Prescriptions/Nurse **	Nurse Prescriptions		Prescribing Nurses		Mean Prescriptions/Nurse
Month	N	%	N	%		N	%	N	%	
January	176	0.1	43	1.2	4	30,173	5.3	2828	50.8	11
February	1034	0.7	115	3.2	9	33,169	5.8	3150	56.6	11
March	2122	1.5	236	6.5	9	43,601	7.6	3435	61.7	13
April	2815	2.0	370	10.3	8	39,623	6.9	3508	63.1	11
May	5317	3.8	613	17.0	9	45,132	7.9	3759	67.6	12
June	7077	5.1	813	22.6	9	49,033	8.5	3900	70.1	13
July	9952	7.1	1054	29.2	9	49,922	8.7	3809	68.5	13
August	11,856	8.5	1249	34.7	9	44,794	7.8	3547	63.8	13
September	17,698	12.7	1957	54.3	9	52,333	9.1	3971	71.4	13
October	24,231	17.4	2541	70.5	10	54,518	9.5	4062	73.0	13
November	28,078	20.1	2608	72.4	11	66,766	11.6	4238	76.2	16
December	29,079	20.9	2679	74.3	11	64,758	11.3	4252	76.4	15
Total Catalonia	139,435	100	3604	-	39	573,822	100	5563	-	103

* The same nurses could issue prescriptions in different months. ** Nurse prescriptions/Prescribing nurses.

**Table 5:** Number of nurse prescriptions and number of nurses that issued prescriptions, by type of product prescribed (2021–2022).

	2021					2022				
	Nurse Prescriptions		Prescribing Nurses *		Mean Prescriptions/Nurse **	Nurse Prescriptions		Prescribing Nurses		Mean Prescriptions/Nurse **
Type of Prescription	N	%	N	%		N	%	N	%	
Materials	101,822	73	2998	83.2	34	376,112	65.5	5001	89.9	75
Medication	37,613	27	2741	76.1	14	197,710	34.5	4761	85.6	42
Total Catalonia	139,435	100	3604	-	39	573,822	100.0	5563	-	103

* A single nurse might have prescribed more than one type of product. ** Nurse prescriptions/Prescribing nurses.

**Table 6:** Number of nurse prescriptions and number of nurses issuing prescriptions for different ATC categories (2021–2022).

	2021					2022				
	Nurse Prescriptions		Prescribing Nurses *		Mean Prescriptions/Nurse **	Nurse Prescriptions		Prescribing Nurses		Mean Prescriptions/Nurse **
ATC Category	N	%	N	%		N	%	N	%	
Nervous system	25,076	66.7	2489	69.1	10	111,524	56.4	4312	77.5	26
Musculoskeletal system	5153	13.7	678	18.8	8	33,059	16.7	2058	37.0	16
Digestive system and metabolism	3190	8.5	789	21.9	4	20,626	10.4	2458	44.2	8
Respiratory system	2641	7.0	371	10.3	7	23,460	11.9	1588	28.5	15
Dermatologic medications	795	2.1	416	11.5	2	3997	2.0	1457	26.2	3
Sensory organs	502	1.3	207	5.7	2	3598	1.8	800	14.4	4
Cardiovascular system	204	0.5	140	3.9	1	1094	0.6	619	11.1	2
Genitourinary system and sex hormones	29	0.1	23	0.6	1	265	0.1	152	2.7	2
Antiparasitics, insecticides, and repellents	8	0.0	6	0.2	1	40	0.0	26	0.5	2
Blood and hematopoietic organs	7	0.0	6	0.2	1	33	0.0	22	0.4	2
Others	8	0.0	5	0.1	2	14	0.0	12	0.2	1
Total Catalonia	37,613	100	3604	-	10	197,710	100	5563	-	36

* A single nurse might have issued prescriptions for >1 ATC category. ** Nurse prescriptions/Prescribing nurses.

**Table 7:** Number of nurse prescriptions and number of nurses issuing prescriptions for different health products (2021–2022).

	2021					2022				
	Nurse Prescriptions		Prescribing Nurses *		Mean Prescriptions/Nurse **	Nurse Prescriptions		Prescribing Nurses		Mean Prescriptions/Nurse **
Material Prescribed	N	%	N	%		N	%	N	%	
Cotton and sterile dressing	52,312	51.4	2570	71.3	20	179,030	47.6	4329	77.8	41
Urine collection kits and devices for urinary and fecal incontinence	25,864	25.4	2259	62.7	11	107,143	28.5	4149	74.6	26
Gauze	10,495	10.3	1758	48.8	6	37,528	10.0	3429	61.6	11
Surgical tape	3991	3.9	1102	30.6	4	13,793	3.7	2368	42.6	6
Bandages	2678	2.6	873	24.2	3	8412	2.2	1923	34.6	4
Ostomy pouching system	1729	1.7	345	9.6	5	9347	2.5	926	16.6	10
Ostomy pouches	1551	1.5	397	11.0	4	6841	1.8	959	17.2	7
Compression socks and stockings	1170	1.1	489	13.6	2	3807	1.0	1373	24.7	3
Urinary catheters, rectal tubes, and gastric tubes	978	1.0	505	14.0	2	3624	1.0	1356	24.4	3
Inhalers and spacers	856	0.8	464	12.9	2	5727	1.5	1611	29.0	4
Knee braces	65	0.1	49	1.4	1	165	0.0	129	2.3	1
Eye patches	45	0.1	37	1.0	1	139	0.0	99	1.8	1
Material for tracheostomies/laryngectomies	34	0.0	19	0.5	2	212	0.1	78	1.4	3
Ankle braces	29	0.0	24	0.7	1	170	0.0	98	1.8	2
Wrist brace	10	0.0	7	0.2	1	97	0.0	59	1.1	2
Truss	7	0.0	6	0.2	1	25	0.0	22	0.4	1
Elbow support	4	0.0	3	0.1	1	14	0.0	7	0.1	2
Jockstrap	2	0.0	2	0.1	1	4	0.0	4	0.1	1
Thigh brace	1	0.0	1	0.0	1	1	0.0	1	0.0	1
Irrigation solution	1	0.0	1	0.0	1	2	0.0	1	0.0	2
Vaginal douche	0	0.0	0	0.0	0	1	0.0	1	0.0	1
Total Catalonia	101,822	100	3604	-	28	376,112	100	5563	-	68

* A single nurse might have issued prescriptions for more than one product. ** Nurse prescriptions/Prescribing nurses.

## Data Availability

Data are contained within the article.
